# Electrocortical activity associated with movement-related fear: a methodological exploration of a threat-conditioning paradigm involving destabilising perturbations during quiet standing

**DOI:** 10.1007/s00221-024-06873-0

**Published:** 2024-06-19

**Authors:** Adam Grinberg, Andrew Strong, Johan Strandberg, Jonas Selling, Dario G. Liebermann, Martin Björklund, Charlotte K. Häger

**Affiliations:** 1 Department of Community Medicine and Rehabilitation, Umeå University, Umeå, Sweden; 2 Department of Statistics, Umeå University, Umeå, Sweden; 3 Department of Physical Therapy, Stanley Steyer School of Health Professions, Faculty of Medical & Health Sciences, Tel-Aviv University, Tel Aviv, Israel; 4 Centre for Musculoskeletal Research, Department of Occupational Health Sciences and Psychology, University of Gävle, Gävle, Sweden

**Keywords:** Re-injury anxiety, Moving platform, EEG, ERP, CNV, Kinesiophobia

## Abstract

Musculoskeletal trauma often leads to lasting psychological impacts stemming from concerns of future injuries. Often referred to as kinesiophobia or re-injury anxiety, such concerns have been shown to hinder return to physical activity and are believed to increase the risk for secondary injuries. Screening for re-injury anxiety is currently restricted to subjective questionnaires, which are prone to self-report bias. We introduce a novel approach to objectively identify electrocortical activity associated with the threat of destabilising perturbations. We aimed to explore its feasibility among non-injured persons, with potential future implementation for screening of re-injury anxiety. Twenty-three participants stood blindfolded on a translational balance perturbation platform. Consecutive auditory stimuli were provided as low (neutral stimulus [CS^–^]) or high (conditioned stimulus [CS^+^]) tones. For the main experimental protocol (Protocol I), half of the high tones were followed by a perturbation in one of eight unpredictable directions. A separate validation protocol (Protocol II) requiring voluntary squatting without perturbations was performed with 12 participants. Event-related potentials (ERP) were computed from electroencephalography recordings and significant time-domain components were detected using an interval-wise testing procedure. High-amplitude early contingent negative variation (CNV) waves were significantly greater for CS^+^ compared with CS^–^ trials in all channels for Protocol I (> 521-800ms), most prominently over frontal and central midline locations (*P* ≤ 0.001). For Protocol II, shorter frontal ERP components were observed (541-609ms). Our test paradigm revealed electrocortical activation possibly associated with movement-related fear. Exploring the discriminative validity of the paradigm among individuals with and without self-reported re-injury anxiety is warranted.

**Supplementary Information:**

The online version contains supplementary material available at 10.1007/s00221-024-06873-0.

## Introduction

The implications of mobility-limiting sports injuries span beyond merely physical and often include psychological consequences which negatively affect subsequent rehabilitation and return to pre-injury level of functioning (Flanigan et al. 2015). Reduced confidence in one’s physical abilities can lead to either conscious or unconscious fear-avoidance behaviour (Quinn and Fallon 1999), with re-injury worries being one of the most commonly cited reasons for not returning to sports after injury (Kvist et al. 2005; Ardern et al. 2014). A distinction between fear and anxiety (specifically, state anxiety) may be appropriate in this context but is seldom made in the orthopaedic literature. Fear generally relates to a present, clearly identifiable threat. In the context of re-injury, fear may be experienced upon the perception that a dangerous movement is about to occur and would potentially cause re-injury. On the other hand, anxiety is a more long-term, future-oriented state (American Psychological Association 2023) and could potentially increase the likelihood and intensity of fear responses during threatening situations. Re-injury anxiety has been defined as “Worries over the possibility of an injury recurring after an initial injury of the same type and location” (Walker and Thatcher 2011). In this sense, this sort of anxiety does not align with a clinical disorder as outlined in the diagnostic and statistical manual of mental disorders (DSM-IV). It is instead a term used in sports psychology and relates to the subjective evaluation of physical danger associated with recurrent injury (Pijpers et al. 2005). Re-injury anxiety has been hypothesised to induce a more internal focus on whole-body movements (Wulf and Lewthwaite 2009). Specifically, preoccupation with physical sensations arising from an injured body part may result in an athlete perceiving these sensations as signs of imminent injury (Andersen and Williams 1993). This has been suggested to lead to diminished biomechanical efficiency and decreased attention to performance-related details (Anders et al. 2008), which by itself may be a risk factor for re-injury (Wulf and Prinz 2001). Indeed, self-reported re-injury worries have been found to be manifested in physical characteristics such as landing biomechanics (Trigsted et al. 2018; Markström et al. 2021), as well as being predictive of secondary injuries (Tagesson and Kvist 2015; Paterno et al. 2018). Since such psychological barriers are modifiable, the development of accurate screening tools may facilitate the identification of individuals at risk and thus improve rehabilitation outcomes. Currently available tools are restricted to subjective self-reported measures and are therefore not necessarily sensitive to unconscious fear/anxiety-related physiological behaviour that may be exhibited during sporting activities (Pijpers et al. 2005).

To our knowledge, objective tools to assess post-injury fear/anxiety do not currently exist. However, movement-related fear can potentially be approximated by exposing individuals to a relevant provocation (i.e., stimuli associated with threatening movements) and measuring the consequential arousal response. Event-related potentials (ERP) are widely used as neural correlates to various psychological and cognitive constructs (Luck 2014). Several ERP components have been suggested as neural correlates to fear of pain (Zheng et al. 2018), fear of spiders (Kolassa et al. 2005; Schienle et al. 2008) and of snakes (Miltner et al. 2005). Invoking these fears typically involves presenting participants with aversive stimuli relevant to the measured construct (e.g., pictures of painful situations, spiders or snakes) (Schienle et al. 2008; Scharmüller et al. 2011). The concept of movement-related fear, often referred to as kinesiophobia (Kori 1990), may require alternative test paradigms where an actual movement-related threat is introduced. The contingent negative variation (CNV) is a slow cortical negative wave that has been associated with anticipatory attention and motor preparation (Brunia and Van Boxtel 2001; Brunia et al. 2012). It has also been observed as a form of a conditioned response to fear and disgust stimuli (Wang et al. 2022), as a neural correlate reflecting anxiety-dependent changes in cognitive preparation to quick motor responses (Proulx and Picton 1984; Ansari and Derakshan 2011) and as pre-emptive anticipatory reaction to expected balance perturbations (Jacobs et al. 2008). In the current study, we introduce an experimental paradigm in which we invoke a conditioned (arousal) response to a threatening situation using sudden perturbations in the form of translations of the base of support. The primary aim of this study was to assess the ability of this experimental test paradigm to measure an electrocortical anticipatory response associated with an impending movement-related threat, among non-injured physically active young individuals. Further, as this approach involved an extensive exposure to repeated stimuli and perturbations, we also addressed the possibility of boredom or potential habituation that might attenuate cortical processing over time.

## Methods

### Participants

Twenty-three healthy individuals (nine females) participated (Table 1). As physically active participants were sought, recruitment was done via advertisement in local sports clubs as well as convenient samples within the university. Inclusion criteria were: 18–35 years of age, right-hand preference for writing and a knee-specific physical activity level of moderate or greater (Tegner activity score (Tegner and Lysholm 1985) ≥ 4). Individuals were excluded if they had suffered any orthopaedic/neurologic pathology in the preceding year or an earlier injury with residual symptoms limiting mobility. Potential participants were also screened for low knee-related confidence (i.e., to what extent did they trust their knee), as we hypothesised that this could influence their perceived levels of threat. For this screening, the Knee injury and Osteoarthritis Outcome Score (KOOS) (Roos et al. 1998) was administered prior to testing. Question 3 of the Quality-of-Life domain – *“How much are you troubled with lack of confidence in your knee?”* – was used as an independent measure of knee-related confidence (Hart et al. 2014; Skou et al. 2015). All participants provided prior written informed consent according to the declaration of Helsinki and the study was approved by the Swedish Ethical Review Authority (Dnr 2021–03860).

**Table 1:** Participant characteristics. Values are presented in mean (SD) unless stated otherwise

	Protocol I		Protocol II
N	23		12
Sex, M/F	13/10		7/5
Age, years	24.4 (4.0)		23.7 (3.4)
Body height, cm	174.5 (9.1)		175.7 (7.5)
Body mass, kg	73.4 (13.4)		72.6 (10.7)
BMI, (kg/m^2^)	24.0 (3.1)		23.6 (3.3)
^a, b^ Tegner activity score (0–10)	7.2 (4–10)		7.0 (4–10)
^a, c^ KOOS, Quality of life, Q3 (%)*	100 (50–100)		100 (50–100)

BMI, body mass index; KOOS, knee osteoarthritis outcome score; Q3, question 3

^a^ Values are presented as median (minimum-maximum)

^b^ Higher scores represent greater level of physical activity

^c^ Higher scores represent greater knee confidence

* Further information regarding other KOOS subscales is available as supplementary material (Table S-1)

### Experimental design

**Fig. 1:**
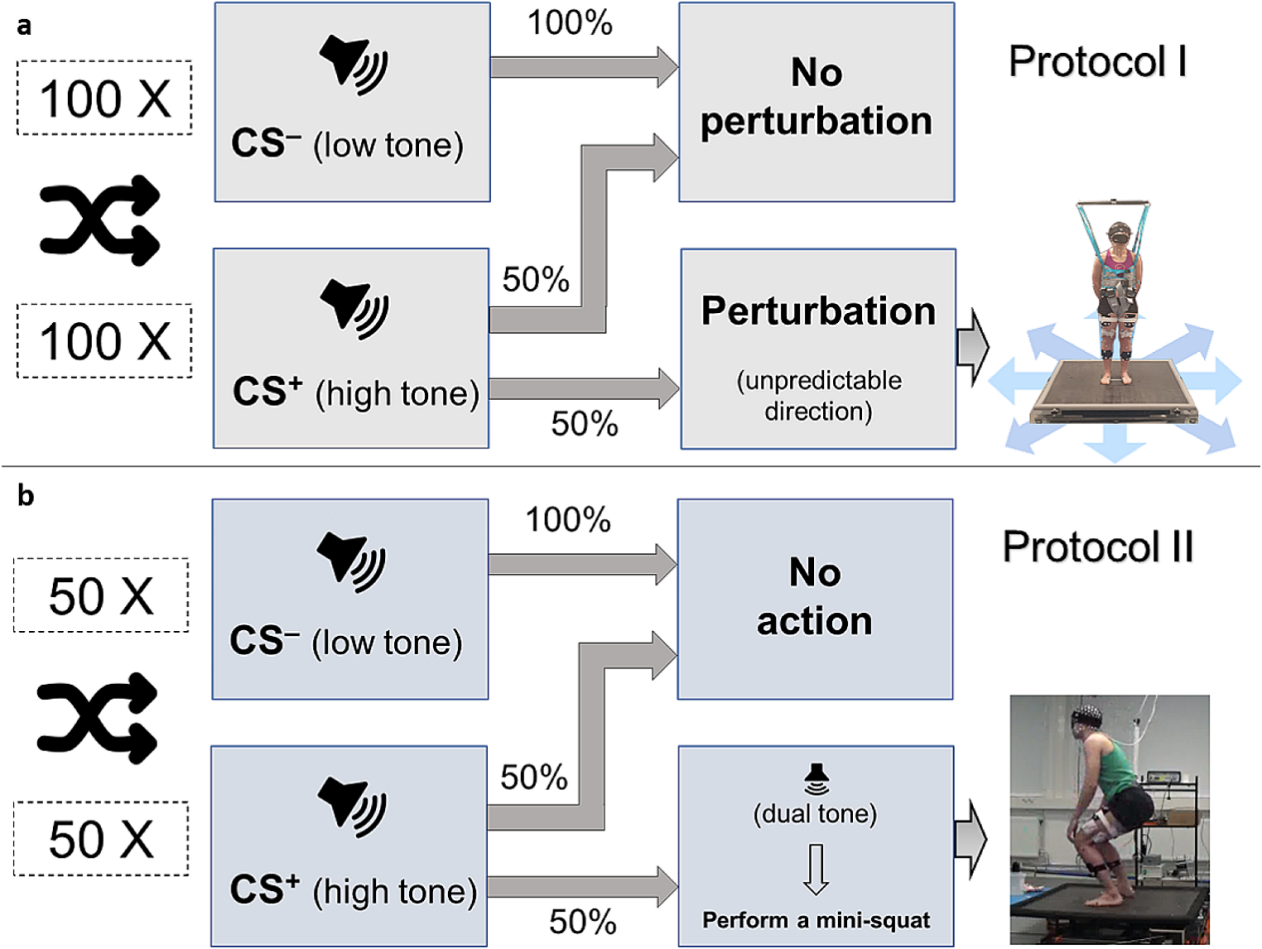
Experimental protocols. (**a**) Protocol I (Fear conditioning) – Blindfolded participants were standing on our platform capable of generating high-acceleration translations in eight directions. Consecutive auditory cues (CS^–^ [Neutral stimulus] or CS^+^ [conditioned stimulus] were presented. The CS^+^ trials were followed by a perturbation half of the time to an unexpected direction. (**b**) Protocol II (Validation protocol [motor response without fear]) – Following a CS^+^, a dual tone was heard half of the time, in which case participants were instructed to react by performing a mini-squat. CS, conditioned stimulus

Testing took place at the U-Motion laboratory, Umeå university, Sweden. The procedure involved a threat conditioning protocol (Protocol I) and a validation protocol (Protocol II). For Protocol I (Fig. 1A), a custom-built, spring-based perturbation platform was used. The platform generated high-acceleration translations (~ 14 m/s^2^, 15 cm) in eight different directions (forward, backward, left, right and diagonals). A safety harness (ErgoTrainer, Ergolet, DK) was used during the test to prevent participants from contacting the floor in case of a fall but provided no support in an upright standing position.

Participants stood blindfolded, hands behind their back holding a rope and in a narrow-base stance on the middle of the platform. To relocate to the starting position in the middle of the platform following postural adjustments due to perturbations, a 1.5 mm thick rope was taped centrally on the surface of the platform in a rectangular shape (28 cm x 28 cm). Participants were thus able to feel this prominence with their feet while blindfolded. This setup was used to increase the difficulty of the task and thus potentially induce a stronger fear response. Participants were also instructed to stand still and particularly to minimise head movements to avoid artefacts in the measured brain response. No additional instructions were given with regards to posture. During the test, a series of consecutive auditory stimuli (at four-second intervals) were automatically generated using customised software integrated with the platform. Either low or high tones were presented in a pseudo-randomised order with an equal probability and corresponded to the following two task conditions:

1. CS^–^: a low tone (1000 Hz) with no following perturbation.
2. CS^+^: a high tone (3000 Hz) with a 50% chance of being followed by a sudden perturbation in either of eight directions, 1.5 s after the tone.

The conditions were explained to the participants prior to the test but the exact probability was not revealed. Participants were only informed that there was a chance of a sudden movement after the high tones. Participants were instructed to maintain their balance and encouraged to minimise excessive limb movements (e.g., stepping). However, no strict limitations were imposed and it was emphasised that they could step if required and were encouraged not to rely on the safety harness (“pretend the harness is not there”). This was stressed to increase the threat of a loss of balance and to reduce the likelihood of habituation after repeated trials. Following a perturbation, a 15-second interval allowed participants to find their centre frame of reference, as the platform slowly returned to its centre position.

Prior to Protocol I, four familiarisation trials were performed: CS^–^ trial followed by CS^+^ trial, repeated twice. Both familiarisation CS^+^ trials were followed by perturbations to opposite directions. Protocol I was then performed and included 100 x CS^–^ and 100 x CS^+^ trials, divided equally into two blocks (~ 15 min each). Rest between blocks of approximately 5 min was included.

Following 5 min of rest, twelve participants (five females) then performed Protocol II (Fig. 1B), which involved one additional test block without perturbations. The rationale was to include a similarly designed protocol with an imperative stimulus that required a comparable whole-body motor preparation (Yazawa et al. 1997), but without a fear element. To achieve this, CS^+^ trials – again indicated by a high tone – were followed by a double tone (2700 Hz), 1.5 s following the initial tone, in 50% of the trials. After each double tone, participants were instructed to perform a “mini-squat”, as quickly as possible. One 100-trial block was performed, with an equal number of CS^–^ and CS^+^ trials.

### Data acquisition and pre-processing

EEG data were recorded using a dense 64-channel HydroCel Geodesic Sensor Net and a Net Amps 400 amplifier (Electrical Geodesic Inc., Eugene, Oregon, USA). Data were sampled at 1000 Hz, referenced to the vertex electrode (Cz) and continuously digitised with a 24-bit analog-to-digital converter. Impedances were verified to be below 50kΩ prior to each test block. All pre-processing was performed using the EEGLAB toolbox (Delorme and Makeig 2004), the ERPLAB extension (Lopez-Calderon and Luck 2014) and custom MATLAB scripts (MathWorks, US). Bad channels were detected via visual inspection and handled by applying spherical interpolation. Trials containing perturbations were identified and a linear replacement was performed between 1000-21000ms after the tone (starting from the mean value 100ms prior to the replacement period to mean 100ms after). This was done to remove transients that could spread into the analysed segments when applying a filter. A finite impulse response band-pass filter (0.1–25 Hz) was then applied, and continuous data were segmented into 1000ms epochs, starting from 200ms before stimulus. Baseline correction was performed using the mean of the 200ms intervals before stimulus onset as reference. Data were then re-referenced to the mastoid average (Luck 2014) and nine electrodes were then selected for the analysis: Frontal (F3, Fz, F4), Central (C3, Cz, C4) and Parietal (P3, Pz, P4) scalp locations (Kolassa et al. 2005; Schienle et al. 2008). Bad-trial detection was performed using a joint probability method (Hu and Zhang 2019), with local (within channel) and global (between channels) rejection thresholds of 3xSD (Demchenko et al. 2016). All the remaining trials were visually inspected following initial automatic rejection for additional artifacts, with all decisions for manual rejection made without knowledge of the trial condition (Luck 2014). Participants retained on average 88.5% ± 4.2 CS^–^ and 89.6% ± 4.5 CS^+^ trials in Protocol I and 88.7% ± 6.2 CS^–^ and 91.7% ± 3.3 CS^+^ trials in Protocol II. Next, an extended independent component analysis (ICA) was performed on epoched data to further detect non-brain independent components (IC) (Onton et al. 2006). The ICLabel tool (v1.3) in EEGLAB was then used as a basis for initial automatic classification of ICs (Pion-Tonachini et al. 2019). Only ICs labelled as functional brain components with > 75% probability (Sherman et al. 2023) were retained and included in the trial averaging, following further verification by means of visual inspection of components’ ERP waveforms, power spectra and scalp topography. Mean probability of brain classification for each participant’s retained IC was 95.6 ± 3.2% for Protocol I trials and 93.9 ± 3.2% for Protocol II trials. The ERPLAB extension was then used for averaging the trials and calculating ERP waveforms and scalp topographies.

### Statistical analysis

An initial detection of between-condition differences was performed using an interval-wise testing (IWT) procedure (Pini and Vantini 2017) applied to average ERP waveforms for latencies 0-800ms after the auditory cue, on all nine channels independently from one another. The procedure, relying on permutation tests, provides an adjusted p-value function for each function which is corrected for multiple testing, and thus enables identification of intervals with statistical significance between the two EEG signal means. The statistical tests were carried out separately for Protocol I and II based on the paired difference waves resulting from subtracting the average EEG response of the CS^–^ from the CS^+^ signal for each individual. For Protocol I, time effects (e.g., habituation) were evaluated for each of the conditions on an individual level by comparing the first 20 trials with the last 20 trials using IWT. For Protocol I only, discrete analyses were performed on the computed mean amplitude of each electrode across significant intervals to detect the most significant channel for identifying condition differences. For all comparisons, individual variance was controlled for by using the difference waves (i.e., CS^–^ – CS^+^). Data were checked for normal distribution using the Shapiro Wilk test and histograms and assessed for extreme outliers using boxplots, with values > 3 X interquartile range excluded from the analysis. Following this inspection, two participants were removed from the discrete analysis for demonstrating extremely high values for both CS^–^ trials, CS^+^ trials and the differences. Data were then submitted to a 3 × 3 repeated measures analysis of variance (ANOVA) with two within-subject factor categories: Caudality (frontal, central, parietal) and Laterality (left, central, right). Bonferroni corrections were performed for pairwise post-hoc comparisons. R version 3.6.1.(Team 2019) and the Statistical Package for the Social Sciences software (version 25, IBM SPSS statistics, Armonk, New York, USA) were used for the main statistical analysis. Statistical significance level was set to 0.05 for all comparisons.

## Results

### Functional data – conditional differences

**Fig. 2:**
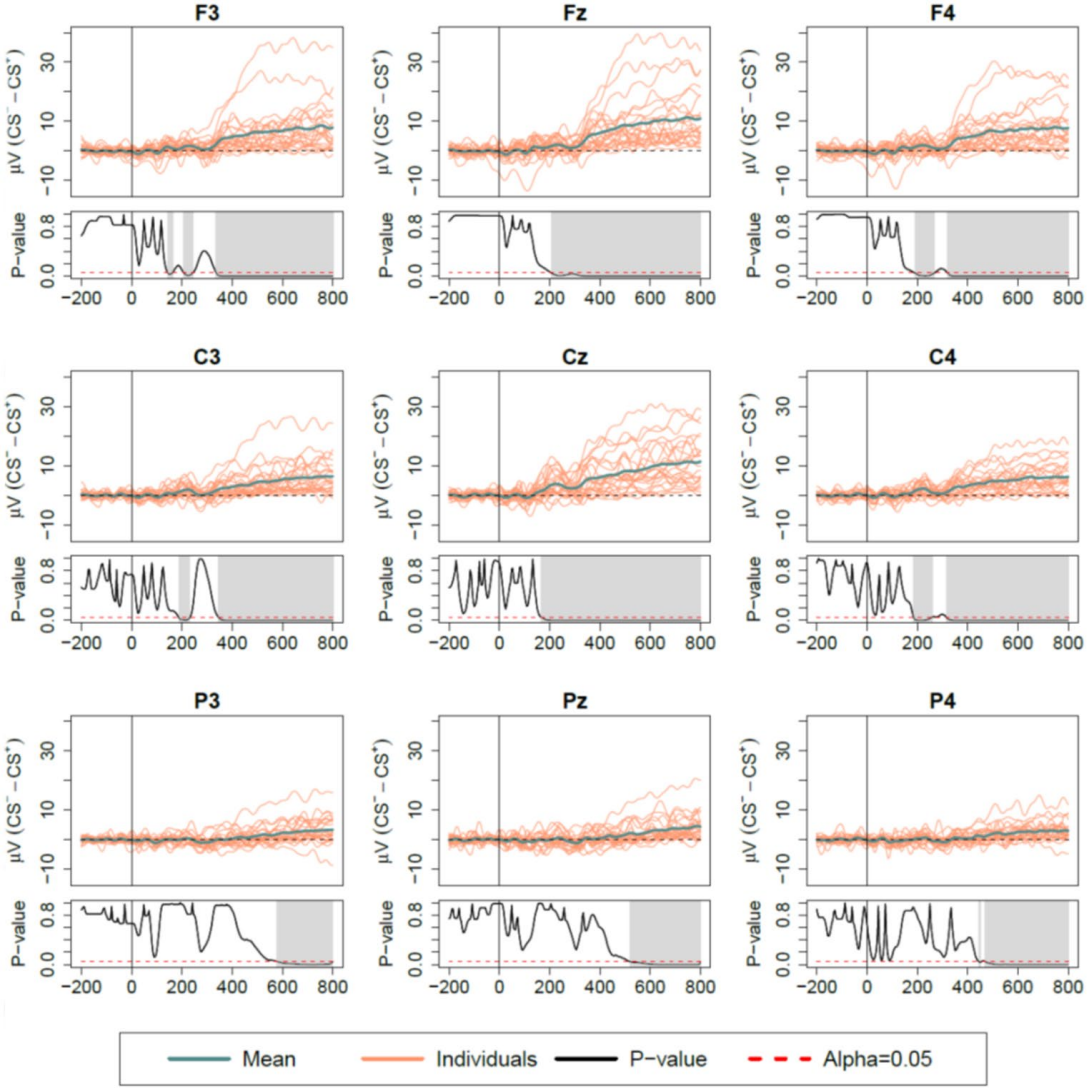
ERP difference waves (CS^–^ – CS^+^) for Protocol I (fear conditioning). Intervals with significant condition effect are highlighted for each channel in corresponding p-value function plots. Time 0 marks the point of auditory cue. ERP, event-related potentials; CS, conditioned stimulus

**Fig. 3:**
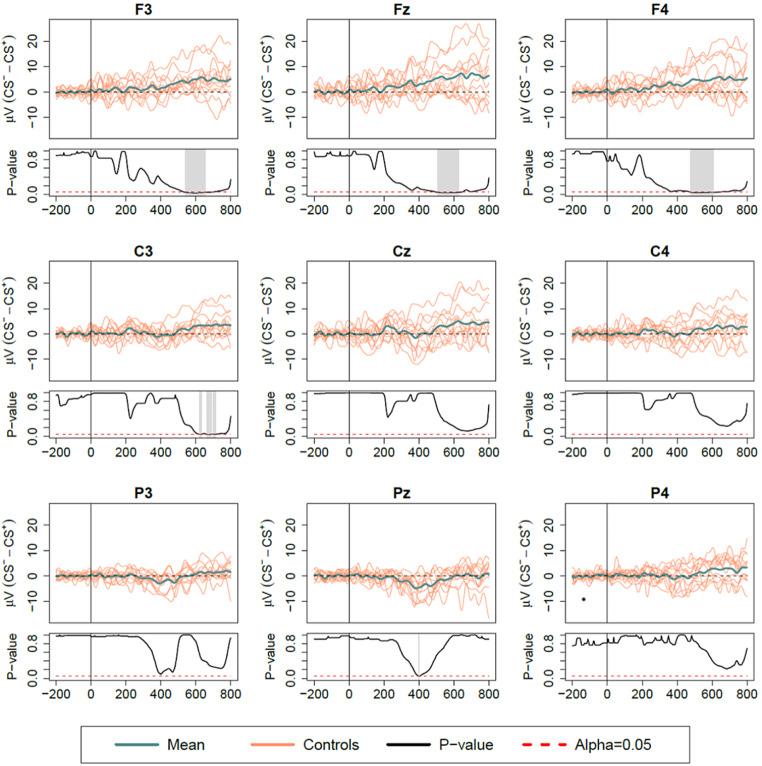
ERP difference waves (CS^–^ – CS^+^) for Protocol II (validation trials – motor response without fear). Intervals with significant condition effect are highlighted for each channel in corresponding p-value function plots. Time 0 marks the point of auditory cue. ERP, event-related potentials; CS, conditioned stimulus

Grand-average waveforms (difference-waves) are shown in Figs. 2 and 3 for Protocols I and II, respectively. A distinct CNV wave was observed with higher amplitude for CS^+^ compared to CS^–^ trials in both protocols, although differences extended over longer intervals and starting from earlier latencies in Protocol I, compared to Protocol II.

In Protocol I, significant latencies from > 344-800ms were observed for all frontal and central electrodes, with mean p-value < 0.001, as well as earlier significant latencies observed shortly around 200ms (mean p-value = 0.011). For all parietal electrodes, significant intervals were observed from > 521-800ms (mean p-value = 0.012).

In Protocol II, significant latencies for CS^–^ – CS^+^ comparisons were mostly observed for frontal electrodes, particularly in latencies 541-653ms (mean p-value = 0.038), 505-629ms (mean p-value = 0.041) and 477-609ms (mean p-value = 0.044), for F3, Fz and F4, respectively. Additional short latencies were observed for C3 electrode around 621-714ms (mean p-value ≤ 0.048). Altogether, these differences suggest that the observed CNV was particularly prominent during threat conditioning CS^+^ trials rather than when a mere motor response was required.

### Discrete statistics – threat conditioning (protocol I) channel effect

**Fig. 4:**
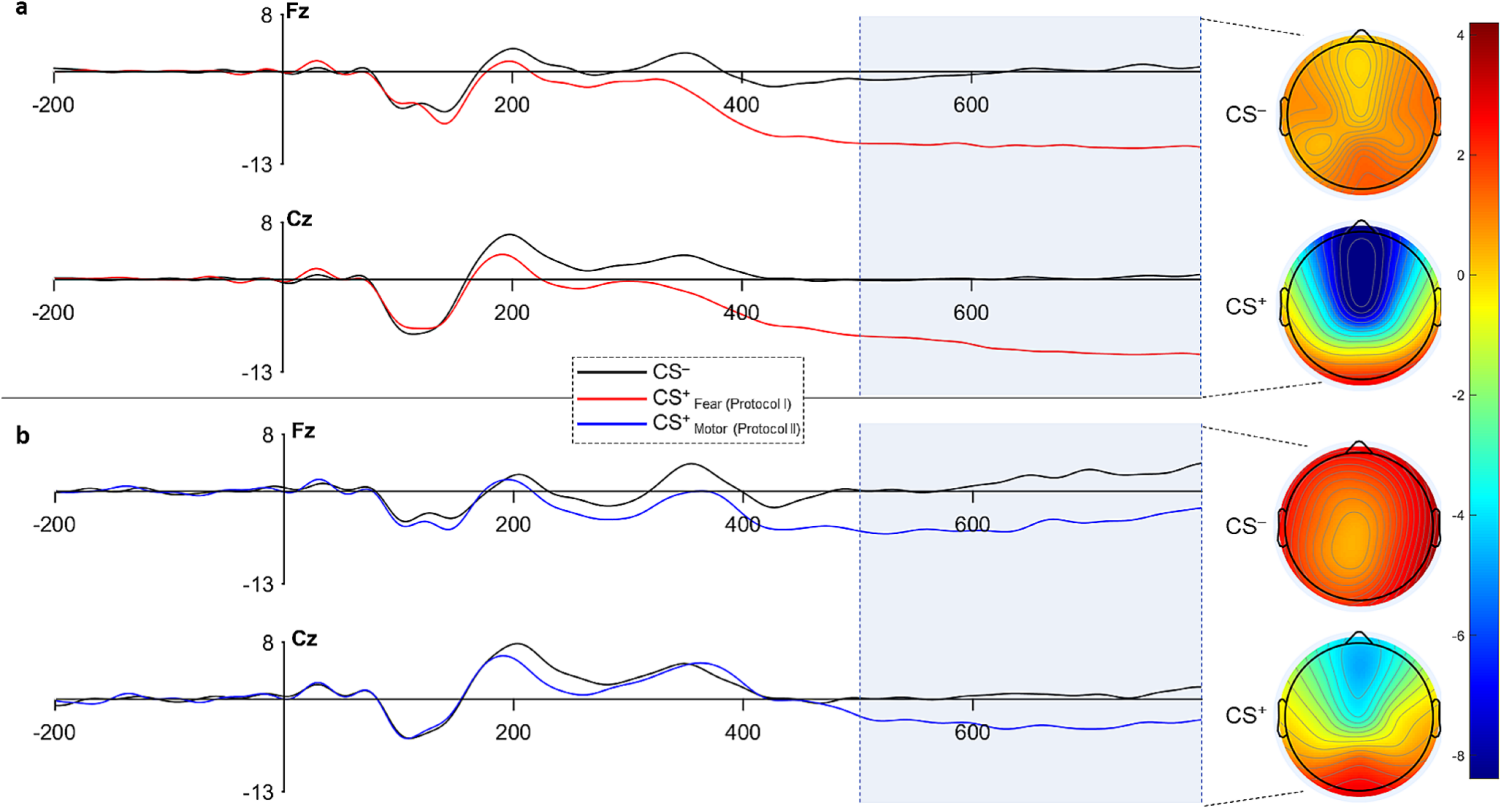
ERP grand averages for electrodes Fz and Cz, presented for Protocol I (fear conditioning; (**a**) and Protocol II (validation trials – motor response without fear (**b**). Scalp topographies are presented as mean amplitude for latency 500-799ms. Time 0 marks the point of auditory cue. ERP, event-related potentials; CS, conditioned stimulus

For all nine channels, signal amplitudes from intervals 500 to 799ms were averaged for discrete comparisons. Significant effects were observed for Caudality (F(2,19) = 21.43, *P* < 0.001) and Laterality (F(2,19) = 12.89, *P* < 0.001). Post-hoc comparisons revealed smaller condition differences across parietal compared to both frontal and central channels and across midline compared to right and left channels (*P* < 0.001 for all comparisons; Table 2). Grand-average ERP waveforms for channel Fz and Cz and scalp topographies for 500-799ms are shown in Fig. 4.

**Table 2:** Protocol I. (fear conditioning) mean ERP amplitudes (µV; mean (SD)) from 500 to 799ms

		CS^–^	CS^+^	Difference
Frontal	F3	-1.04 (3.60)	-6.17 (4.28)	5.13 (4.09)
	Fz	-1.88 (3.49)	-10.01 (5.40)	8.13 (5.90)
	F4	-0.93 (2.83)	-6.69 (3.33)	5.76 (4.22)
Central	C3	0.08 (2.43)	-4.22 (4.09)	4.30 (3.32)
	Cz	-0.50 (3.18)	-8.71 (5.92)	8.22 (5.94)
	C4	0.25 (2.20)	-4.38 (3.52)	4.63 (3.66)
Parietal	P3	-0.04 (2.79)	-1.83 (3.37)	1.78 (2.26)
	Pz	0.88 (2.61)	-1.43 (2.99)	2.31 (2.33)
	P4	0.38 (2.29)	-1.65 (2.31)	2.04 (1.66)

ERP, event-related potentials; CS, conditioned stimulus

### Time effect

**Fig. 5:**
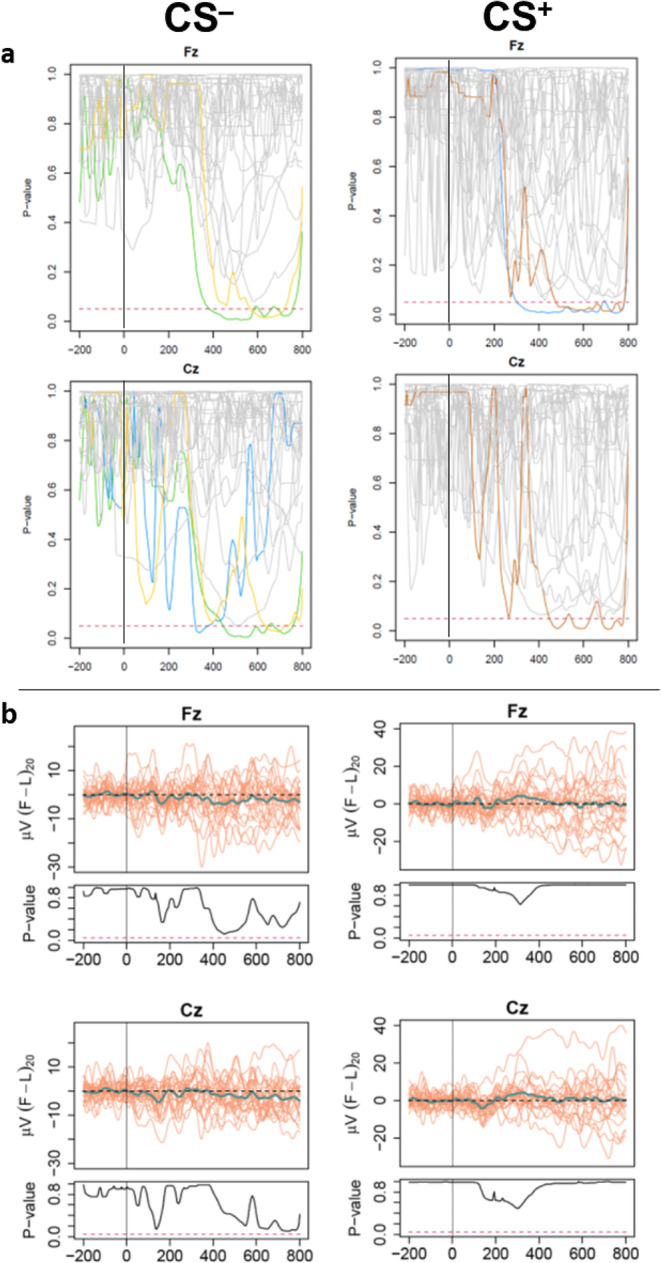
Individual comparisons of the first and last 20 trial average differences [(F – L)_20_], presented for electrodes Fz and Cz, separately for CS^–^ and CS^+^ trials. Time 0 marks the point of auditory cue. (**a**) Individual p-value functions with *p* = 0.05 indicated by a dashed line. Participants with significant time differences are presented in distinct colours. Otherwise, presented in grey. (**b**) Group averages. CS, conditioned stimulus

Comparisons between the first and last 20 trials for each individual are presented in Fig. 5 for selected channels (Fz, Cz). On an individual level, four participants per condition demonstrated significant differences between their first and last 20 ERP responses (Supplementary figures S1, S2, for CS^–^ and CS^+^, respectively). However, due to a lack of consistent findings (i.e., some presented higher, and others presented lower average late response compared to the average early response), we chose to base all analyses on all available data. A group-level analysis confirmed that there were no significant time effects in either of the conditions (Supplementary figures S3, S4, for CS^–^ and CS^+^, respectively).

## Discussion

The possibility of a forthcoming perturbation during CS^+^ trials induced a significantly larger CNV amplitude compared to CS^–^ trials that were knowingly not proceeded by a perturbation. This was observed for all nine electrode cites, with the most prominent effect in central and frontal midline locations. In contrast, a shorter CNV response was observed, albeit inconsistently, when motor preparation was required without threat of perturbation. Furthermore, the lack of differences in response intensity between the first and last trials indicate that participants did not habituate to the repeated threat.

### From threat to expectation to fear

The CNV wave was originally addressed in the context of anticipation (Walter et al. 1964). It is generally elicited after a stimulus provided within a certain timeframe prior to a potential second stimulus that requires a motor reaction (Van Boxtel and Böcker 2004). As the CNV is associated with individuals waiting for an imperative stimulus to follow, it is commonly referred to as an “expectancy wave” (Brunia and Van Boxtel 2001). In the present study, participants were assured that no perturbation would follow the low tone in the CS^–^ trials. This presumably eliminated an anticipation response compared to CS^+^ trials. In that sense, our study follows a classic expectancy experimental design with a CS^+^ introducing threat and invoking anticipation to a necessary motor response. In contrast to classic designs, however, our required motor response involved whole-body adjustments rather than a simple response such as pushing a button (Walter et al. 1964). Our study is comparable to previous work by Jacobs et al. (Jacobs et al. 2008), who reported pre-perturbation CNV responses in anticipation to an upcoming externally-triggered backward translation of the support surface. Notably, Protocol I introduced unpredictability in the required balance response due to the random direction of the perturbations. This greater unpredictability potentially heightened proactive processes of anticipatory error detection and conflict monitoring that occur even before the actual ‘error’ or perturbation happens. Such processes are known to involve activation of the cingulate cortex (Carter et al. 1998; Botvinick et al. 2004), potentially influencing CNV amplitudes considering previously identified activity in the cingulate gyrus that coincides with CNV generation (Nagai et al. 2004). In contrast, the relative predictability of the required response in Protocol II suggests a minimal level of anticipatory error monitoring, resulting in negligible CNV differences between conditions. We do acknowledge that this anticipatory error monitoring is distinct from fear, which is more related to an emotional reaction to the potential of an upcoming perturbation. An anticipatory motor reaction which does not match the direction of the perturbation could nevertheless result in loss of balance and potential stress on passive anatomical structures. The current paradigm could thus theoretically elicit a certain fear response, rather than mere expectancy, although this cannot be confirmed based solely on our data. It has previously been noted that despite reflecting a controlled contingency estimation, the CNV wave may be influenced by the affective quality of the eliciting stimulus (Regan and Howard 1995). Activity in the cingulate gyrus is known to be modulated by uncertainty and arousal (Critchley et al. 2001), both of which were potentially enhanced during CS^+^ trials in Protocol I due to the heightened anticipation. Along with the insula, which has also been implicated in CNV paradigms (Nagai et al. 2004), a meta-analysis of fMRI studies found that these cortical areas are activated during fear conditioning paradigms as part of a large-scale fear-related brain functional network (Fullana et al. 2016). The cingulate gyrus has also established anatomical connections with the orbitofrontal cortex, a region associated with reinforcement learning (Rushworth et al. 2007) and which has a distinct role in modulating emotions during fear conditioning paradigms (Tabbert et al. 2005). The CNV wave is estimated to have further origins in the pre-motor cortex (Hultin et al. 1996) and supplementary motor areas (Nagai et al. 2004), which are also implicated in an extended functional ‘fear network’ (Fullana et al. 2016). In the current study, the necessity of whole-body adjustments suggests that the observed CNV may be an indication of increased pre-motor activation required for coordinating and planning complex movements (Ghez 2000). It is also well known that the amygdala is another brain structure linked to fear conditioning (Tabbert et al. 2005; Öhman 2009). While reportedly not a region involved CNV generation (Nagai et al. 2004), the amygdala is known to be vastly interconnected with the orbitofrontal cortex and other prefrontal areas, forming a functional network for emotional processing (Barbas 2007). As source localisation was not within the scope of the current study, the involvement of an extensive fear network remains hypothetical. Interestingly, the insula, which also contributes to CNV generation (Nagai et al. 2004), has been shown to be activated by the mental recall of fearful experiences (Damasio et al. 2000). While this was most likely not the case in the present study due to the lack of such an experience among non-injured participants, it may be of relevance among injured individuals who may consequently demonstrate stronger ERP responses.

### Fear and other ERP responses

While other ERP components have been linked to certain fears, such as the P200 (Zheng et al. 2018), P300 (Baas et al. 2002; Miltner et al. 2005) and the late positive potential (LPP) (Kolassa et al. 2005; Miltner et al. 2005; Schienle et al. 2008; Zheng et al. 2018), non was observed in the present study to differentiate between conditions. It is not uncommon that smaller components are masked by larger-amplitude components that have an overlapping latency (Luck 2014). The CNV in our data was observed from latencies of 300ms in some channels, thus overlapping with commonly observed P300 and LPP latencies (Hajcak and Foti 2020). Furthermore, P300 and LPP commonly reach a maximum amplitude in parietal-occipital and central-parietal recording cites, respectively (Hajcak and Foti 2020). Our results conversely demonstrate larger conditional differences seen around centre-midline and frontal locations, which is consistent with CNV-observed areas (Falkenstein et al. 2003). The unconditioned stimulus in our test paradigm was also inherently different than studies focusing on e.g., fear of spiders and its expression in P300 and LPP amplitudes (Miltner et al. 2005; Schienle et al. 2008). In those experiments on phobic participants, ERPs were elicited in response to the presentation of aversive pictures. An electrocortical response was thus evoked presumably based on previous beliefs and image-associations rather than actual threat. In a recent study by An et al. (An et al. 2019), individuals with anterior cruciate ligament (ACL) injury and non-injured controls were presented with visual stimuli of neutral, general-fear and injury-relevant pictures. Both the injury-relevant and general-fear/aversive pictures stimuli induced a similar cortical effect for both groups. It is thus somewhat unclear whether indirectly referring to injury-relevant movements using images could trigger mental recall of personal experiences manifested in an arousal response. Alternate paradigms may be required if cortical responses to movement-related fear are to be elicited. Conversely, our paradigm included a tangible threat, which may also explain why ERP components were dissimilar than those elicited from aversive pictures (Miltner et al. 2005; Schienle et al. 2008). In another study, Baas et al. (Baas et al. 2002) applied electric shocks to induce an arousal response to actual threat. Although eliciting pain is inherently different from the unconditioned stimulus in our paradigm, threat-conditioning was common to both. Similarly to our study, Baas et al. observed a frontal negative slow wave associated with the threat of an electric shock compared to no threat.

### Implications and future directions

Re-injury fears and anxieties are considered hindrances for injured persons who aim to return to physical activity and are reported as one of the most common reasons that athletes fail to return to competitive sports (Kvist et al. 2005; Ardern et al. 2014). High-anxiety individuals are also hypothesised to be more internally focused during performance (i.e., focused on their injured body part) (Heil 1993; Moran 2013), which is a precursor for decreased performance (Wulf et al. 2001) and may predispose them to secondary injuries (Wulf and Prinz 2001). However, commonly administered subjective tools are not necessarily optimal to capture precise anxiety levels (Grinberg 2023). The Tampa scale of kinesiophobia (TSK), for example, was originally designed for pain-dominating musculoskeletal problems (Miller et al. 1991), yet remains one of the most commonly used tools to assess fear of movement following sports injuries (Norte et al. 2019; Bakhsh et al. 2021). Due to its origin, the TSK focuses mainly on pain as a dominating consequence of excessive movement (Miller et al. 1991). The suitability of the TSK among populations in which pain is no longer a disturbing issue is however unclear (Ohji et al. 2021). Although more suitable questionnaires are in use, e.g., the ACL return to sports after injury (ACL-RSI) (Webster and Feller 2018), a potential problem with all self-reported outcome measures is the likelihood of social desirability bias to contaminate questionnaire responses (King and Bruner 2000). This is particularly a risk when assessing sportspeople who may wish to present themselves favourably to comply with sport-relevant demands, during various opportunities for self-promotion (Grossbard et al. 2007). Our current paradigm conversely invoked actual movement-related threat, which could theoretically complement questionnaire-based inquiries as part of a holistic investigation of re-injury anxiety experienced by injured persons. Our paradigm should next be further tested among injured individuals with higher levels of self-reported re-injury worries, to validate the clinical significance of the measured brain response. Incidentally, while the focus of our study is primarily sport-related injuries among athletes, the concepts of movement-related anticipation, fear and anxiety are relevant to other populations such as elderly persons, people who have had a stroke and those with chronic pain. Future studies are encouraged to explore this line of research across a broader spectrum of demographics and patient groups. Moreover, future clinical evaluations may incorporate similar tests using more portable dry-electrode EEG systems (Lopez-Gordo et al. 2014). Such systems, although more prone to movement artefacts, have previously been shown to successfully measure laboratory-quality ERP waveforms and scalp topographies, particularly for large components (Mathewson et al. 2017).

### Limitations

While our paradigm seemed to invoke a conditioned arousal response, the question remains as to whether fear was elicited. Additional work should assess its construct validity with subjective outcomes related to anxiety and fear. Such outcomes include measures of self-reported fear of movement (Lundberg et al. 2004), psychological readiness to return to sport (Webster and Feller 2018) or self-efficacy (Thomeé et al. 2006), all constructs which may be associated with re-injury anxiety. No such assessments were used in the current study since existing assessments tools are based on the psychological response to an injury and thus irrelevant for non-injured persons. We did include a single question regarding knee confidence from the KOOS questionnaire, but a larger sample size (particularly low-confidence individuals) is likely required to achieve sufficient power for discriminative purposes. Secondly, as already mentioned, we did not perform source space localisation of the observed response, which prohibits any direct determination of fear-related cortical generators. This should be explored in future studies to further confirm a relationship between the measure threat-related heightened anticipation and fear-related brain structures. Thirdly, previous work indicates that females and males demonstrate differences in emotional regulation in general, and EEG responses in particular (Peng et al. 2023). Considering this, we ran the discrete analysis with sex as a covariate, which did not influence the significance level of our results. We also performed the analysis with sex as between-subjects factor and found no significant differences between males and females. We nevertheless acknowledge that sex differences may be a legitimate concern when applying the perturbation paradigm on individuals with self-reported re-injury anxiety. In this sense, future implications should consider sex as a potential confounder. Fourthly, while no postural instructions were given, preparatory strategies involving lowering of the centre of mass following high tones (e.g., slightly flexing the knees) were observed in some participants, while presumably supressed or otherwise absent in others. This could suggest better perceived readiness among those who displayed greater postural preparation (Rabbani et al. 2014), which could potentially influence their cortical response. While this consideration was of less importance in the current study, future similar investigations including group comparisons should consider such potential behavioural confounders. Lastly, it has been shown that cortical processing may be different for low compared with high frequency sounds (Pratt et al. 2009). In that respect, a certain difference between test conditions, attributed to the dissimilarities between the two tones, cannot be entirely excluded.

## Conclusions

Our test paradigm successfully captured an electrocortical response to the threat of sudden destabilising perturbations. A CNV wave was elicited when a risk of a sudden movement was introduced, potentially indicative of threat-induced cortical activation, implying heightened anticipation, error monitoring and arousal. Further, despite the long experimental procedure, participants did not seem to habituate. In a separate validation protocol, a CNV wave was observed more sparsely and over shorter delayed intervals when a motor response was required without perturbation, further supporting the involvement of anticipatory error monitoring and a potential emotional component in the main protocol. Future studies should further confirm the role of fear in such a response, as well as explore the discriminative ability of such an approach between individuals with and without previous musculoskeletal injury and with different levels of self-reported re-injury anxiety.

### Electronic supplementary material

Below is the link to the electronic supplementary material.

Supplementary Material 1

## Data Availability

The datasets generated during and/or analysed during the current study are available from the corresponding author on reasonable request.
