# Supplementary material for: Electrocortical activity associated with movement-related fear: a methodological exploration of a threat-conditioning paradigm involving destabilising perturbations during quiet standing
Source: Exp Brain Res. 2024 Jun 19;242(8):1903–15. doi: 10.1007/s00221-024-06873-0 (PMC11252179; doi:10.1007/s00221-024-06873-0)
Supplement: Supplementary file 1 — Supplementary Material 1 [file 221_2024_6873_MOESM1_ESM.pdf]

Supplementary material (Protocol I time effect – extended results)

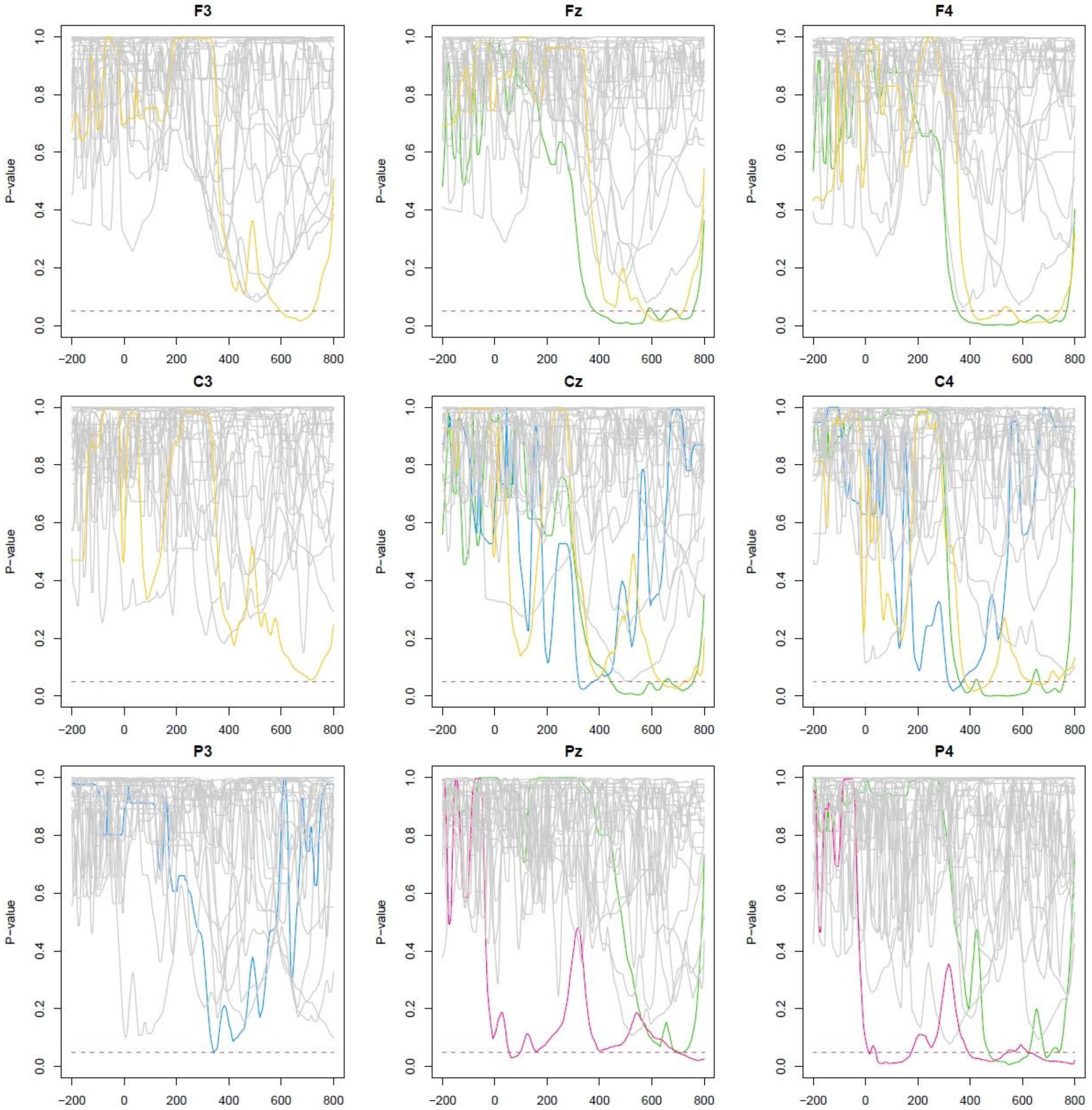

Figure S1. Individual p-values for first and last 20 trials average – *CS^{-} trials.* Significance level of p = 0.05 is indicated by a dashed line. Participants with significant time differences are presented in distinct colours. Otherwise, presented in grey.

ERP, event-related potentials; CS, conditioned stimulus

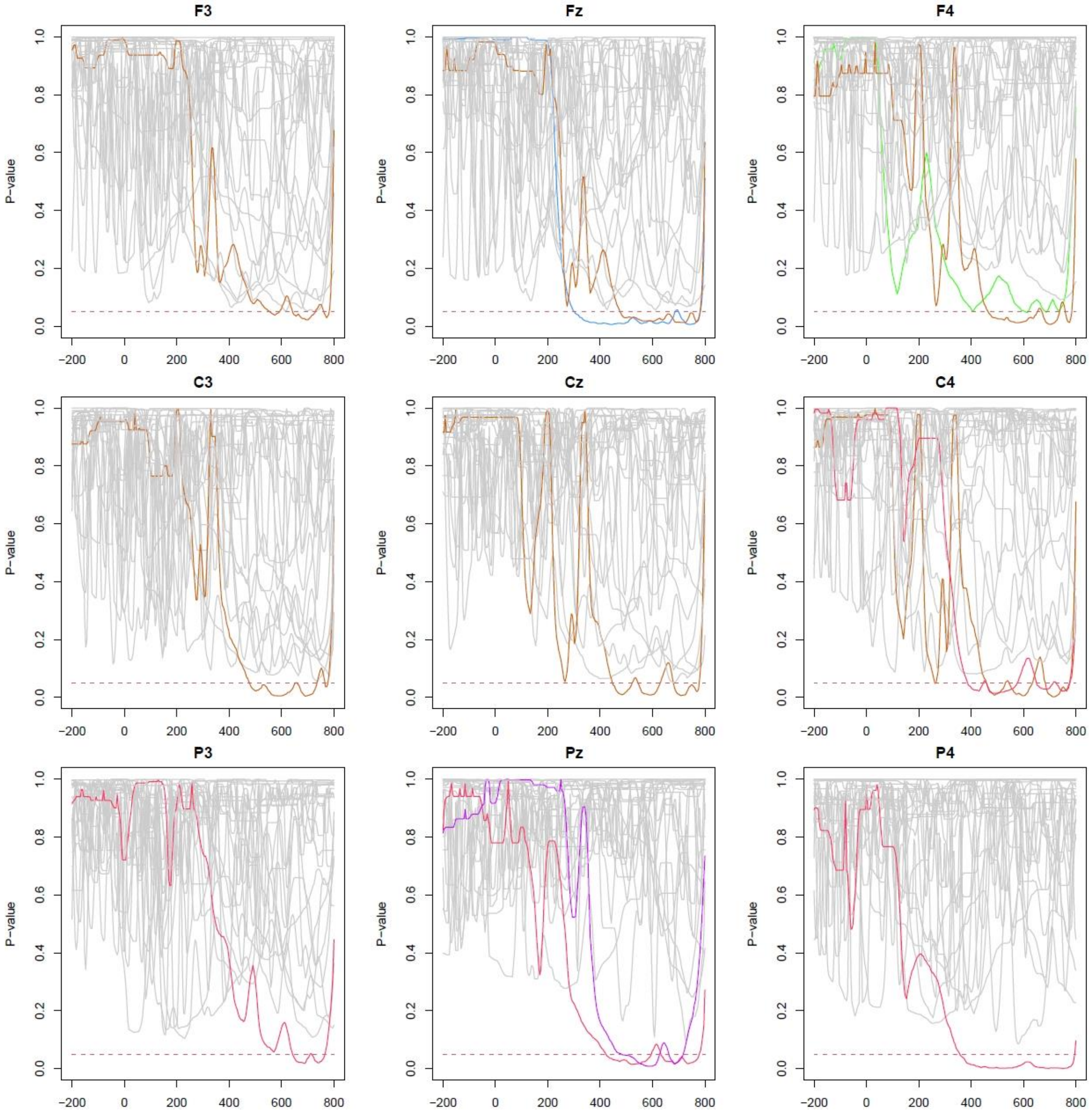

Figure S2. Individual p-values for first and last 20 trials averages – *CS⁺ trials.* Significance level of p = 0.05 is indicated by a dashed line. Participants with significant time differences are presented in distinct colours. Otherwise, presented in grey.

ERP, event-related potentials; CS, conditioned stimulus

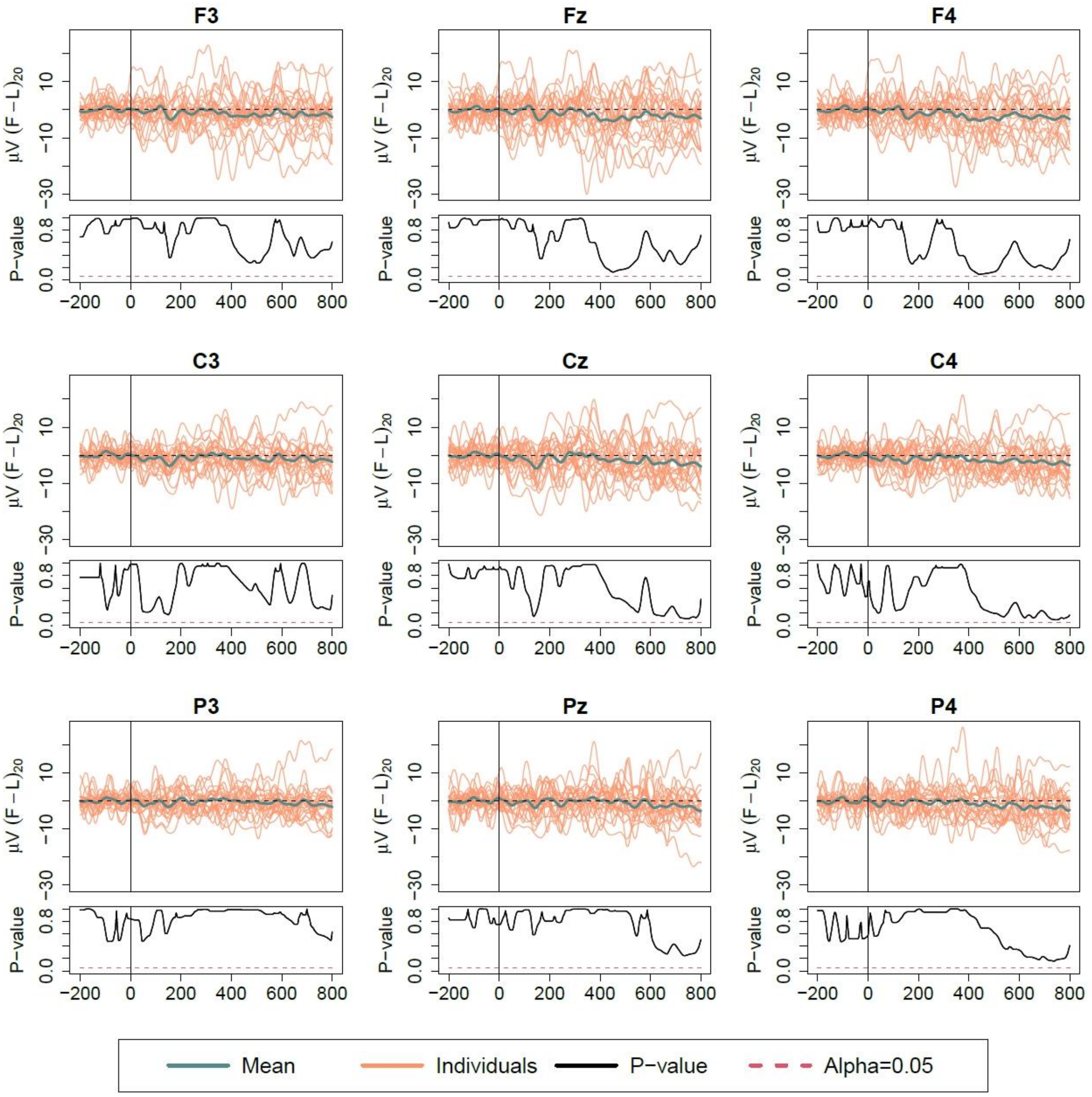

Figure S3. Group comparison for individual time effects by comparing the first and last 20 trial average differences $[(F - L)_{20}] - CS^{-}$ *trials.*

ERP, event-related potentials; CS, conditioned stimulus

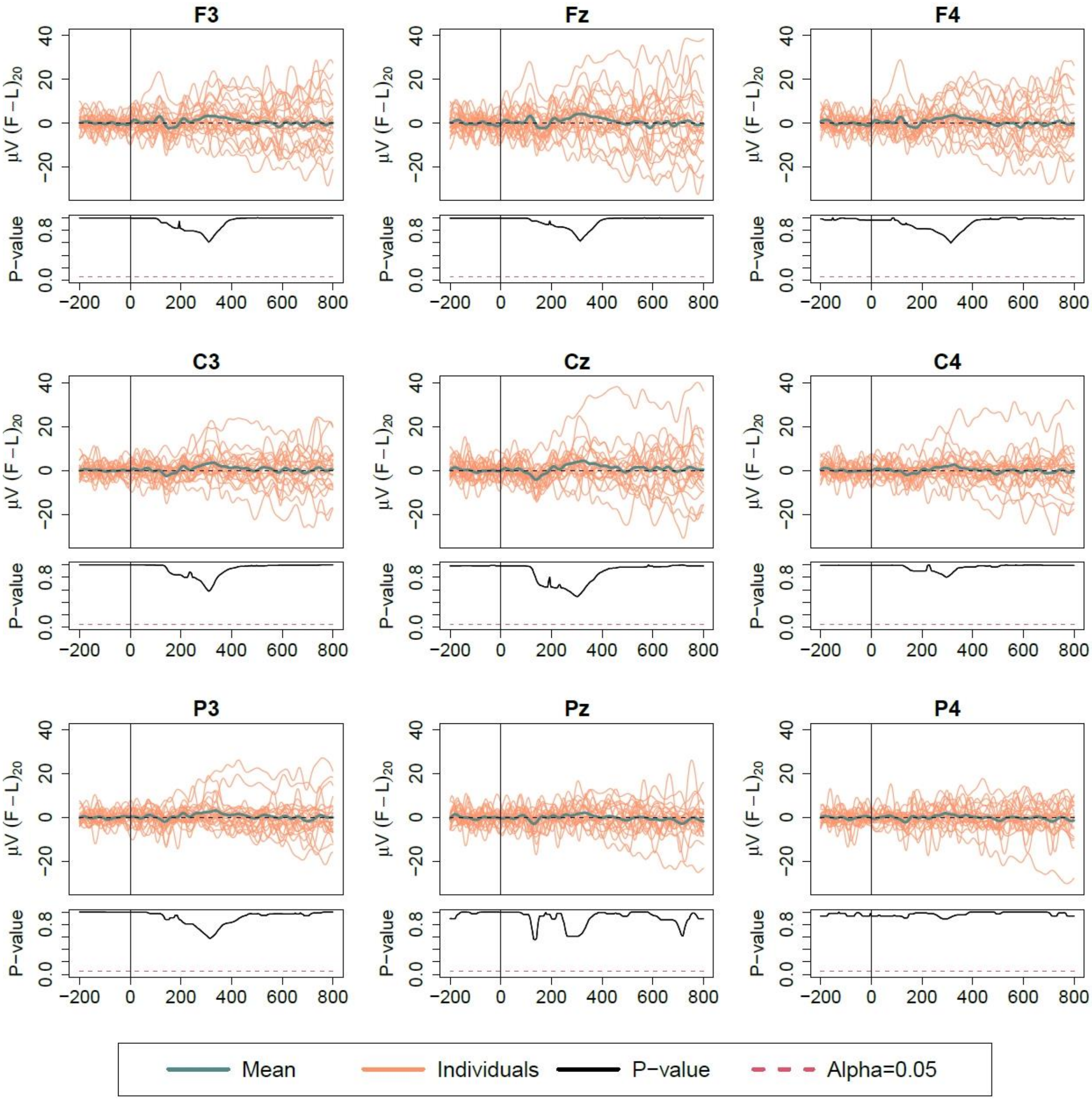

Figure S4. Group comparison for individual time effects by comparing the first and last 20 trial average differences $[(F - L)_{20}] - CS^{+}$ *trials.*

ERP, event-related potentials; CS, conditioned stimulus

Table S-1 Knee osteoarthritis outcome score (KOOS) values for the study participants.

**KOOS subscale (%)***	**Protocol I (n=23)**	**Protocol II (n=12)**
Symptoms	96 (68-100)	98 (82-100)
Pain	100 (81-100)	100 (81-100)
Activities of daily living	100 (74-100)	100 (74-100)
Sports and recreation function	100 (75-100)	100 (75-100)
Quality of life	94 (69-100)	97 (69-100)

*Values are presented as median (min-max).
